# REGEN-COV as the First Line of Defense—A Single-Centre Experience

**DOI:** 10.3390/life16010074

**Published:** 2026-01-04

**Authors:** Milica Popović, Vladimir Đurović, Bojana Ljubičić, Nadica Kovačević, Slobodan Šajinović, Lada Petrović, Tatjana Ilić, Sonja Golubović

**Affiliations:** 1 Faculty of Medicine, University of Novi Sad, 21000 Novi Sad, Serbia; milica.popovic@mf.uns.ac.rs (M.P.); bojana.ljubicic@mf.uns.ac.rs (B.L.); nadica.kovacevic@mf.uns.ac.rs (N.K.); slobodan.sajinovic@mf.uns.ac.rs (S.Š.); lada.petrovic@mf.uns.ac.rs (L.P.); tatjana.ilic@mf.uns.ac.rs (T.I.); sonja.golubovic@mf.uns.ac.rs (S.G.); 2 Clinic of Nephrology and Clinical Immunology, University Clinical Centre of Vojvodina, 21000 Novi Sad, Serbia; 3 Department of Emergency Internal Medicine, Emergency Centre, University Clinical Centre of Vojvodina, 21000 Novi Sad, Serbia; 4 Clinic of Infectious Diseases, University Clinical Centre of Vojvodina, 21000 Novi Sad, Serbia; 5 Centre for the Transplantation of Organs, Cells, and Tissues, University Clinical Centre of Vojvodina, 21000 Novi Sad, Serbia

**Keywords:** COVID-19, monoclonal antibody, mortality

## Abstract

Background: Casirivimab–imdevimab (REGEN-COV) is a neutralizing antibody cocktail that has been shown to prevent the progression of COVID-19 and serious adverse outcomes in patients with COVID-19 disease. During the period preceding the emergence of the Omicron variant, REGEN-COV demonstrated clinical activity against several circulating SARS-CoV-2 variants. The delta variant was dominant worldwide during much of the study period. Aim: This retrospective observational study aimed to show the single centre’s results in treating patients with REGEN-COV. Methods: This study included adult patients who received REGEN-COV at our COVID-19 centre from 01 June 2021 to 31 January 2022 (REGEN-COV group) and a comparison group that included patients who did not meet the eligibility criteria for REGEN-COV (non-REGEN-COV group). The primary end-point was the need for hospitalization. The secondary end-point was all-cause mortality. Intensive care unit admission was also evaluated. Results: This study included 206 patients, of whom 69 received REGEN-COV, and 137 comprised the non-REGEN-COV group. During follow-up, 128 patients (62%) required hospitalization, including 11 (15.9%) in the REGEN-COV group and 117 (85.4%) in the non-REGEN-COV group (*p* < 0.001). Mortality occurred in 2 patients (2.9%) treated with REGEN-COV compared with 30 patients (21.9%) in the non-REGEN-COV group. In the propensity score-matched analysis, the average marginal absolute risk difference for mortality between the groups was −4.0% (95% CI −9.8 to 1.8), *p* = 0.178; however, Kaplan–Meier survival analysis demonstrated a significant difference in survival between groups (log-rank *p* < 0.001). Conclusions: Our study showed that in high-risk patients, with specific variants of SARS-CoV-2, the use of REGEN-COV was associated with a lower risk of hospitalization, and it was associated with better disease outcomes. One of the limitations of this study was the variant-specific effectiveness of REGEN-COV, which may limit generalizability.

## 1. Introduction

Severe Acute Respiratory Syndrome coronavirus 2 (SARS-CoV-2) causes coronavirus disease 2019 (COVID-19), and up until June 2024, it had affected more than 775 million people [1]. Most of those affected by COVID-19 are outpatients, but in some cases, the progression of the disease leads to hospitalization or even death [2]. During successive pandemic waves, mortality rates varied substantially across regions and time periods. Data from the United States showed that the cumulative number of COVID-19 deaths peaked in late 2020, followed by subsequent peaks in mid-2021 and early 2022 [3]. Mortality rates were even more pronounced in upper- and lower-middle-income countries compared with high-income countries [4]. The clinical presentation and disease progression of COVID-19 were highly heterogeneous. Moreover, the circulation and successive emergence of SARS-CoV-2 variants during the pandemic were associated with differences in transmissibility, virulence and response to available therapies, and this contributed to variability in clinical outcomes across time [5].

Neutralizing monoclonal antibodies are derived from the B cells of convalescent patients or humanized mice. These antibodies have specificity and affinity to bind to a virus and block the entry of the virus, therefore abrogating a pathology associated with productive infection. The primary antigenic epitope on SARS-CoV-2 is the spike (S) protein, responsible for target cell binding and fusion upon engaging cell surface angiotensin-converting enzyme 2 receptors. These receptors are found on cells in the respiratory system, gastrointestinal tract and endothelium. Consequently, antibodies directed to the S protein can neutralize the ability of the virus to interact with the target host cell [6]. Anti-SARS-CoV-2 spike protein-neutralizing monoclonal antibodies have shown in vivo efficacy in animal models, with decreases in viral load and lung pathology [7,8].

Early into the pandemic, passive antibody-based therapies were explored as a potential treatment option for individuals with COVID-19, since these therapies proved to be successful in previous coronaviruses outbreaks [9]; however, their efficacy ranged from no effect on the outcome to an improved outcome with or without late complications [10]. These observations, nonetheless, provided an important biological rationale for the development and early use of standardized neutralizing monoclonal antibodies in COVID-19, particularly in vulnerable populations at increased risk of disease progression. Early antibody-based interventions were hypothesized to be the most effective before extensive inflammatory lung involvement occurred, bringing the relevance of treatment timing into focus [11]. In the United States, three anti-SARS-CoV-2 neutralizing monoclonal antibodies received emergency use authorization for the treatment of non-hospitalized patients with mild-to-moderate COVID-19 disease. These included bamlanivimab as a monotherapy and the combination therapies bamlanivimab–etesevimab and casirivimab–imdevimab. While this authorization enabled the clinical use of these therapies, their real-world utilization was subject to several limitations including complex production processes, potentially limited supply and uncertainty regarding the duration of their protective effect [6].

Casirivimab–imdevimab (REGEN-COV) is a neutralizing antibody cocktail that acts against the spike protein of SARS-CoV-2 [12,13,14]. In vitro studies showed that the administration of REGEN-COV reduces viral loads and allows its activity to be retained against the alpha, beta, gamma, delta, epsilon and iota variants of the virus. REGEN-COV showed no efficacy against the Omicron variant of SARS-CoV-2 [15,16]. Data from phase 3 clinical trials further demonstrated that the early use of REGEN-COV in high-risk outpatients can lower the risk of hospitalization or death from any cause [14]. REGEN-COV acquired its first emergency use licence in the United States in November 2020, and the European Union followed in February 2021 [17].

The aim of this study was to evaluate clinical outcomes associated with REGEN-COV therapy compared with the standard of care in a real-world setting.

## 2. Materials and Methods

### 2.1. Study Design

This retrospective observational study was conducted among adult patients who were diagnosed with COVID-19 disease at the Clinical Centre of Vojvodina, Novi Sad, from the 1st of June 2021 to the 31st of January 2022. All patients included in this study were considered high-risk patients due to an underlying disease, old age or obesity. No patients required supplemental oxygen therapy or hospitalization due to COVID-19 during the first evaluation at our centre. The demographic and clinical information evaluated included age, gender, an individual medical condition that qualified the patient to receive REGEN-COV, vaccinal status, level of inflammatory markers (C-reactive protein and procalcitonin) and the presence of incipient pneumonia.

All included patients had to be over 18 years of age and had to have a positive SARS-CoV-2 polymerase chain reaction or antigen test. Patients were divided into two groups depending on whether they had received REGEN-COV or not. Patients entitled to the drug had to have mild to moderate symptoms of COVID-19 and were within 5 days of symptom onset. Additionally, at least one of the following criteria had to be met in order for individuals to be classified as high-risk and be eligible for the drug: presence of chronic kidney disease, immunocompromising condition, diabetes mellitus, obesity (defined as BMI ≥ 35 kg/m^2^) or immunosuppressive drug use, for age ≥ 65 years, or if ≥ 55 years, presence of chronic lung disease, hypertension or cardiovascular disease [14]. In the REGEN-COV group, the drug was administered at a dose of 1200 mg intravenously (600 mg of both casirivimab and imdevimab), followed by a 2 h observation period. The non-REGEN-COV group included high-risk patients who were not eligible to receive the drug either because the disease lasted longer than 5 days or they refused the REGEN-COV treatment. Patients in the non-REGEN-COV group were treated according to the usual standard of care which included corticosteroids, low-molecular-weight heparin and gastric ulcer prophylaxis.

Patients above 80 years old, pregnant individuals and those who needed a higher level of oxygen therapy at the disease presentation were excluded from the analysis.

All patients were followed for 90 days, and the primary end-point was the need for hospitalization due to disease progression. The secondary end-point was all-cause mortality. The rates of intensive care unit (ICU) admission and the use of mechanical ventilators were also evaluated.

### 2.2. Statistical Analysis

The normality of continuous variables was assessed using the Kolmogorov–Smirnov test. Non-normally distributed continuous variables were reported as the median with the interquartile range (Q1–Q3). Continuous variables were compared between independent groups using the Wilcoxon rank-sum test, while categorical variables were compared using the chi-square test or Fisher’s exact test, as appropriate. To address baseline differences between treatment groups, propensity score matching was applied. Propensity scores were estimated using a probit regression model with treatment assignment as the dependent variable and baseline covariates including age, sex, vaccination status, pneumonia at presentation, hypertension and diabetes mellitus. Full matching yielded the best balance. After matching, all standardized mean differences (SMDs) for the covariates were below 0.1, indicating adequate balance, as shown in Table 1. Full matching used all treated and non-treated units, so no units were discarded by the matching. Treatment effects were estimated in the matched sample using a weighed generalized linear model with a binomial distribution. The primary estimand was the average marginal effect of treatment on mortality, expressed as an absolute risk difference. Kaplan–Meier curves were used to visualize survival probabilities, with weighted estimates based on propensity score full matching. All statistical tests were two-tailed, and an alpha level of 0.05 was set as a significance threshold. No imputations were used for the missing data. Statistical analyses were conducted using RStudio 2025.05.0+496 “Mariposa Orchid” Release.

## 3. Results

Overall, this study included 206 patients with a median age of 64 (51–71) years, and 120 (58%) were males (Table 1). Seventy-eight (38%) patients were vaccinated. Of all patients, 69 received REGEN-COV. Propensity score full matching retained all 206 individuals for further analyses.

When comparing the two groups, the REGEN-COV group included younger individuals (median age 58 (45–71) vs. 65 (54–71), *p* = 0.037); however no age differences were present after matching (SMD < 0.1). Additionally, no differences were observed in gender distribution before (*p* = 0.339) and after matching (SMD < 0.1). Patients treated with REGEN-COV had an earlier symptom onset (median of 3 [2–4] days) compared with those not treated with REGEN-COV (median of 8 [6–10] days), *p* < 0.001, consistent with the requirement for early presentation for treatment eligibility. Regarding the number of comorbidities and frequency of hypertension, there were no significant differences between the two groups, albeit the non-REGEN-COV group had significantly more patients with diabetes mellitus, and *p* < 0.001 in an unmatched cohort. This difference disappeared after matching was performed. Groups were also well balanced in vaccination status after propensity score matching (Table 1). The most common medical conditions, which were the main reason for the treatment with REGEN-COV, were CKD (29%) and hematological diseases (27.6%); these conditions were not present in the non-REGEN-COV group, as they constituted specific indications for treatment.

**Figure 1:**
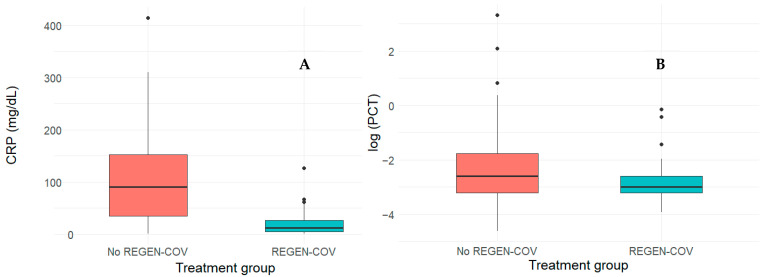
Boxplot showing (**A**) levels of C-reactive protein (CRP, [mg/dL]) of two study groups and (**B**) log-transformed levels of procalcitonin (PCT) of two study groups.

At the initial presentation, patients in the REGEN-COV group had significantly lower C-reactive protein (CRP) levels (median of 12 (5–26) vs. 90 (35–152), *p* < 0.001), while procalcitonin (PTC) levels did not significantly differ (median of 0.05 (0.04–0.08) vs. 0.08 (0.04–0.18), *p* = 0.062), as shown in Figure 1.

One patient (1.4%) developed an infusion-related adverse reaction within one hour after the treatment. The reaction included chills, shivering and fever. After symptomatic therapy, the patient’s discomfort completely resolved. There were no delayed reactions during the follow-up time. Additionally, no hypersensitivity or anaphylactic reactions were observed in the treatment group.

During the follow-up period, 128 (62%) patients were hospitalized, of which 11 patients received REGEN-COV, while 117 belonged to the non-REGEN-COV group, *p* < 0.001 (Table 2).

Only 1 patient in the REGEN-COV group (1.4%), compared to 24 (18%) non-REGEN-COV patients (*p* < 0.001), was later transferred to the ICU ward. The length of the hospital stay between the groups, however, did not differ significantly, with a median of 11 (9–15) vs. 12 (9–16) days, *p* = 0.932. Serious adverse outcomes were observed in 2 (2.9%) REGEN-COV patients, while 30 (22%) patients died in the non-REGEN-COV group, *p* < 0.001, as shown in Table 3.

Both of the deceased REGEN-COV patients were treated in January of 2022 and were hospitalized due to COVID-19 complications. One patient experienced an exacerbation of symptoms three days following REGEN-COV treatment, needing oxygen therapy and subsequent ICU admission seven days post-hospitalization. Despite being treated with mechanical ventilation, the patient died 42 days after being hospitalized. The second patient was hospitalized five days after REGEN-COV treatment due to persistent coughing. He did not require oxygen supplementation and was discharged from the hospital after ten days. However, ten days post-discharge, he developed symptoms of ileus and died due to complications following surgical intervention, 27 days after the REGEN-COV infusion. At the time of his death, there were no signs or symptoms of COVID-19.

**Figure 2:**
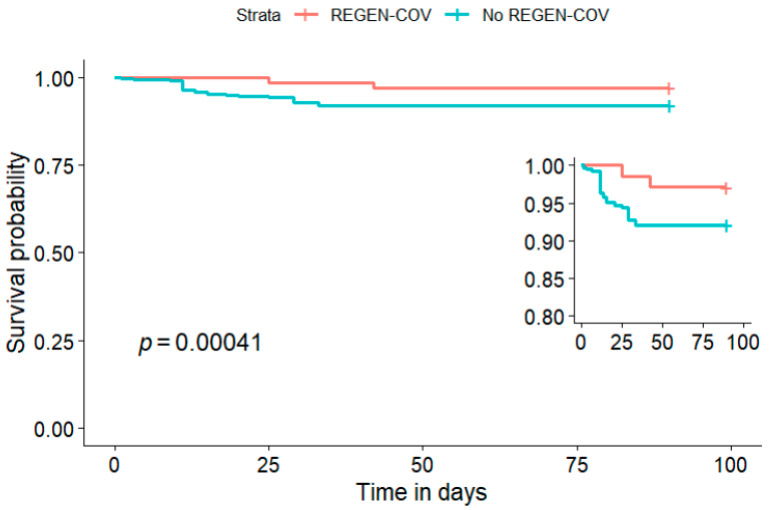
Kaplan–Meier estimate of survival of treated COVID-19 patients stratified by use of REGEN-COV. Analysis was performed using weighted data derived from propensity score full matching. Time to death was assessed over 90-day follow-up period, with patients censored at 90 days if alive. Tick marks indicate censored observations.

In the weighted Kaplan–Maier curve analysis, the REGEN-COV group exhibited significantly better survival compared to the non-REGEN-COV group, with a log-rank *p*-value < 0.001, as demonstrated in Figure 2.

In the propensity score-matched analysis, the average marginal absolute risk difference was −4.0 percentage points (95% CI −9.8 to 1.8); however, this difference did not reach statistical significance, *p* = 0.178.

## 4. Discussion

### 4.1. Demographics

At the University Clinical Centre of Vojvodina, different therapeutic approaches were used to treat patients with COVID-19. From June 2021 to January 2022, we were able to treat the selected population of patients with monoclonal antibodies against SARS-CoV-2. The present study reports the treatment outcomes. Our study included 206 patients with COVID-19 infection, with a median age of 64 (51–71) years, and the majority of patients were male. Out of the entire study population, 69 patients received REGEN-COV, a neutralizing monoclonal antibody cocktail against the spike protein of SARS-CoV-2, which leads to a reduction in viral load and allows its activity to be retained against the alpha, beta, gamma, delta, epsilon and iota variants of the virus [14,15].

### 4.2. Comparison of REGEN-COV and Non-REGEN-COV Groups

In our retrospective observational study, selected patients were divided into two groups, the REGEN-COV and non-REGEN-COV groups, depending on whether they had received a monoclonal antibody or not. Previous studies suggest that the progression of COVID-19 to severe or critical forms is associated with old age; male sex; underlying comorbidities such as obesity, hypertension and chronic pulmonary, kidney, heart and liver diseases; diabetes; immunodeficiency and malignant diseases [18,19,20]. According to these data, we selected a group of high-risk patients, suitable for REGEN-COV treatment. Most patients in the REGEN-COV group were men, with a median age of 58 years, and all of them had at least one medical condition, which, according to current recommendations, categorized them as high-risk for developing COVID-19 complications [18,20,21,22].

After propensity score matching, age was well balanced between groups with a median age of 58 (45–71) years in the REGEN-COV group vs. 65 (54–71) years in the non-REGEN-COV group, SMD < 0.1. No differences were observed in gender distribution, SMD < 0.1. The demographic profile is consistent with previously reported real-world cohorts of patients treated with REGEN-COV [14].

Before matching, the vaccination rate was significantly higher in the REGEN-COV group, *p* = 0.037; however this was balanced out after propensity score matching was applied. Consistent with previous reports, patients in our cohort who were vaccinated against COVID-19 experienced lower rates of hospitalization compared with unvaccinated patients [23,24,25].

There was no significant difference in the number of comorbidities between the two groups (*p* = 0.051), indicating a comparable burden of comorbid conditions at baseline [18,21]. Furthermore, the non-REGEN-COV group had significantly more patients with diabetes mellitus in the unmatched cohort (*p* < 0.001); however the difference was adequately balanced after matching, SMD < 0.1. The most common medical conditions, which were the main reason for the treatment with REGEN-COV, were CKD (29%) and hematological diseases (27.6%), which is similar to previously reported studies [18,21,26,27,28]. One possible explanation for these findings is greater awareness and experience with REGEN-COV therapy among physicians at the University Clinical Centre of Vojvodina in Novi Sad compared with general practitioners managing COVID-19 cases.

All patients in the REGEN-COV group were diagnosed with COVID-19 within 96 h prior to treatment. Patients who received REGEN-COV presented to a physician earlier, with a median of 3 (2–4) days, compared to those who did not receive REGEN-COV, with a median of 8 (6–10) days, *p* < 0.001. Patients suffering from chronic diseases, such as diabetes or hematological or nephrological diseases, who were previously treated at the University Clinical Center, received clear instructions from their attending physicians to report symptoms of COVID-19 infection immediately. The longer duration of the disease in the non-REGEN-COV group posed a contraindication for the treatment with REGEN-COV. Verderese et al. [29] reported a trend toward a higher hospitalization rate with longer delays between symptom onset and REGEN-COV infusion.

A mild form of the disease was present in the entire study group. Signs of incipient pneumonia on chest X-ray were present in sixteen patients (23%), but none required oxygen supplementation at the time of treatment. When compared to the non-REGEN-COV group, there was a significant difference in the prevalence of pneumonia (23% vs. 85%, *p* < 0.001), which balanced out after matching (SMD < 0.1). This discrepancy in the occurrence of pneumonia reflects real-world treatment allocation, as eligibility for REGEN-COV favoured earlier presentation, inherently limiting overlap in pneumonia prevalence between groups.

The serum levels of CRP were markedly higher in the REGEN-COV group (median of 12 vs. 90 mg/dL, *p* < 0.001), whereas PCT levels did not significantly differ (median of 0.05 vs. 0.08, *p* = 0.062). These data suggest that patients in the non-REGEN-COV group presented with a more severe form of the disease, which further contributed to their unsuitability for monoclonal antibody therapy, consistent with the observations reported by Gao et al. [18]. Higher CRP levels may reflect a more pronounced inflammatory response and have been linked to greater disease severity. As such, baseline differences in CRP could act as a confounding factor when interpreting clinical outcomes. In contrast, the absence of differences in procalcitonin values between groups suggests that secondary bacterial infection was unlikely at the time of assessment, which reduces the potential for confounding related to secondary bacterial infection. Although propensity score matching was applied to improve comparability between groups, residual confounding related to disease severity cannot be fully excluded.

All patients in the REGEN-COV group received intravenous REGEN-COV as recommended, and they were followed up for two hours on the day of treatment. There were no serious adverse events related to REGEN-COV administration in our study. We observed only one mild infusion reaction related to the treatment, which subsided completely within a few hours after symptomatic therapy. No hypersensitivity or anaphylactic reactions were observed in the treatment group. There were no delayed reactions within the follow-up. Similar results were found in the available literature [14,30].

After the treatment, patients were followed up for 90 days or to the time of death. We wanted to estimate the risk of hospitalization and fatal outcomes in patients during the period of observation. Previous studies showed that REGEN-COV reduced the need for hospitalization and the rate of lethal outcomes from any cause [14].

For the primary end-point, 128 patients (62%) required hospitalization, most commonly due to COVID-19 progression. Hospitalizations were significantly more frequent in the non-REGEN-COV group compared to the REGEN-COV group (85% vs. 16%, *p* < 0.001), which is consistent with the finding from earlier reports that REGEN-COV is associated with reduced hospitalization rates when given early to high-risk patients [14,31]. The hospitalization rate observed in our REGEN-COV group was higher than previously reported [14,21,28], likely reflecting variant epidemiology during the study window. Although infections were not genotyped, our understanding is that the Omicron variant rapidly became dominant in Serbia in January 2022, and casirivimab–imdevimab has minimal activity against this variant [15,16]. There was no difference in the length of hospitalization between groups, at 11 (9–15) in the REGEN-COV group vs. 12 (9–16) days in the non-REGEN-COV group, *p* = 0.932.

The burden of comorbidity appears to be an important driver of outcomes: we observed that incipient pneumonia on chest radiography, diabetes and hypertension were associated with a higher frequency of hospitalization, which has also been reported previously [32,33].

Importantly, ICU admission was required for only 1 patient who received REGEN-COV (1.4%) vs. 24 (18%) patients that did not receive the drug, *p* < 0.001. All-cause mortality was also significantly lower in the REGEN-COV group than in the non-REGEN-COV group (2.9% vs. 22%; *p* < 0.001). To contextualize the mortality observed in the non-REGEN-COV group, the outcomes of patients with COVID-19 treated at our centre during a comparable period (November 2021 to April 2022) were previously reported by Obradovic et al. who observed an overall mortality rate of 44.8% [34]. While Kaplan–Meier analysis demonstrated better survival in the REGEN-COV group (log-rank *p* < 0.001), the average marginal absolute risk difference did not reach statistical significance (−4.0%, [95% CI −9.8 to 1.8%], *p* = 0.178). Large observational cohorts and pragmatic randomized trials from observational data have reported an association between REGEN-COV treatment and a reduced risk of serious adverse outcomes such as mortality compared with the standard of care. Our findings are directionally consistent with these findings, although the small sample size and limited number of events in our study likely reduced the precision and statistical significance of the estimates [7,12,35,36].

Furthermore, the overall rate of complications and need for escalation of care were lower among patients who received REGEN-COV, similar to the trends reported in some other real-world cohorts [37,38].

### 4.3. Study Limitations

This study had several limitations, including its retrospective, non-randomized design and relatively small sample size. Second, despite the use of propensity score matching, residual imbalance in baseline disease severity cannot be fully excluded, particularly given differences in the timing of presentation and clinical eligibility for REGEN-COV therapy. Third, no viral sequencing was performed; therefore, differences in circulating SARS-CoV-2 variants could not be directly assessed. Finally, untreated patients were enrolled earlier and were likely affected by the different variant of the virus, which may have influenced clinical outcomes.

## 5. Conclusions

Patients treated with REGEN-COV presented earlier and had lower inflammatory burden at baseline. This aligned with treatment eligibility and clinical practice during the study period.

No serious drug-related adverse events were recorded during the study period.

The careful selection of high-risk outpatients and timely administration of REGEN-COV were associated with improved clinical outcomes, including lower rates of hospitalization, ICU admission and mortality. Our findings apply primarily to pre-Omicron variants and may not be generalizable to currently circulating strains.

## Figures and Tables

**Table 1:** Patients’ baseline characteristics.

Parameter	Overall, N = 206 ^1^	REGEN-COV, N = 69 ^1^	Non-REGEN-COV, N = 137 ^1^	*p*-Value ^4^	SMD ^2^
Age (years)	64 (50–71)	58 (45–71)	65 (54–71)	**0.037**	0.0021
Sex (male)	120 (58)	37 (54)	83 (61)	0.339	0.0956
Time from symptom onset to presentation (days) ^3^	6 (3–9)	3 (2–4)	8 (6–10)	**<0.001**	
Vaccinated	78 (38)	32 (46)	45 (33)	**0.036**	−0.0120
Number of comorbidities ≥ 2	143 (69)	54 (78)	89 (65)	0.051	
Diabetes mellitus	37 (17)	1 (1.4)	35 (26)	**<0.001**	0.0175
Hypertension	147 (71)	44 (64)	103 (75)	0.087	0.0696
Pneumonia	133 (65)	16 (23)	117 (85)	**<0.001**	−0.0159

^1^ N (%); median (Q1–Q3). ^2^ SMD—standard mean difference. ^3^ Time from symptom onset to presentation is inherently in the treatment eligibility criteria and was not included in propensity score matching. ^4^ Bolded *p*-values indicate statistical significance (*p* < 0.05). Note: *p*-values are reported descriptively; balance after propensity score matching was assessed using the SMD.

**Table 2:** Comparison of hospitalized and non-hospitalized patients.

Parameter	Overall, N = 206 ^1^	Hospitalized, N = 128 ^1^	Non-hospitalized, N = 78 ^1^	*p*-Value ^2^
Age	64 (50, 71)	66 (54, 71)	60 (42, 71)	**0.015**
Sex (male)	120 (58)	75 (59)	45 (58)	0.899
Vaccinated	78 (38)	39 (30)	39 (50)	**0.005**
Number of comorbidities ≥ 2	143 (69)	87 (68)	56 (72)	0.563
Diabetes mellitus	37 (18)	31 (24)	6 (7.7)	**0.003**
Hypertension	147 (71)	98 (77)	49 (63)	**0.034**
Pneumonia	73 (35)	4 (3.1)	69 (88)	**<0.001**

^1^ N (%); median (Q1–Q3). ^2^ Bolded *p*-values indicate statistical significance (*p* < 0.05).

**Table 3:** Significant outcomes.

Parameter	Overall, N = 206 ^1^	REGEN-COV, N = 69 ^1^	Non-REGEN-COV, N = 137 ^1^	*p*-Value ^2^
Hospitalized	128 (62)	11 (16)	117 (85)	**<0.001**
Length of hospitalization (days)	12 (9–16)	11 (9–15)	12 (9–16)	0.932
ICU ward admission	25 (13)	1 (1.4)	24 (18)	**<0.001**
Death	32 (17)	2 (2.9)	30 (22)	**<0.001**

^1^ N (%); median (Q1–Q3). ^2^ Bolded *p*-values indicate statistical significance (*p* < 0.05).

## Data Availability

The data presented in this study are available upon request from the corresponding author. The data are not publicly available due to them containing information that could compromise the privacy of research participants.

## Abbreviations

The following abbreviations are used in this manuscript:

CKD	Chronic Kidney Disease
BMI	Body Mass Index
ICU	Intensive Care Unit
CRP	C-reactive Protein
PCT	Procalcitonin
